# Is immunosenescence influenced by our lifetime “dose” of exercise?

**DOI:** 10.1007/s10522-016-9642-z

**Published:** 2016-03-29

**Authors:** James E. Turner

**Affiliations:** Department for Health, University of Bath, Claverton Down, Bath, BA2 7AY UK

**Keywords:** Cytomegalovirus, T-lymphocytes, Exercise, Physical activity, Sedentary behaviour, Redox

## Abstract

The age-associated decline in immune function, referred to as immunosenescence, is well characterised within the adaptive immune system, and in particular, among T cells. Hallmarks of immunosenescence measured in the T cell pool, include low numbers and proportions of naïve cells, high numbers and proportions of late-stage differentiated effector memory cells, poor proliferative responses to mitogens, and a CD4:CD8 ratio <1.0. These changes are largely driven by infection with *Cytomegalovirus*, which has been directly linked with increased inflammatory activity, poor responses to vaccination, frailty, accelerated cognitive decline, and early mortality. It has been suggested however, that exercise might exert an anti-immunosenescence effect, perhaps delaying the onset of immunological ageing or even rejuvenating aged immune profiles. This theory has been developed on the basis of evidence that exercise is a powerful stimulus of immune function. For example, in vivo antibody responses to novel antigens can be improved with just minutes of exercise undertaken at the time of vaccination. Further, lymphocyte immune-surveillance, whereby cells search tissues for antigens derived from viruses, bacteria, or malignant transformation, is thought to be facilitated by the transient lymphocytosis and subsequent lymphocytopenia induced by exercise bouts. Moreover, some forms of exercise are anti-inflammatory, and if repeated regularly over the lifespan, there is a lower morbidity and mortality from diseases with an immunological and inflammatory aetiology. The aim of this article is to discuss recent theories for how exercise might influence T cell immunosenescence, exploring themes in the context of hotly debated issues in immunology.

## Introduction and overview

It is well known by the general population, and accepted by research scientists, clinicians and other health professionals, that—although an oversimplification—exercise^1^ is “good for us”. A large number of studies have investigated how lifestyle factors influence the risk of developing disease, affect the treatment of disease, and potentially modify ageing processes. This interest has led to the merging of research areas, once limited to the domain of sport and exercise science, into mainstream biology, medicine and population health. One example is exercise immunology research, which despite landmark findings reported as early as 1893, did not grow in popularity until the 1980s (Shephard 2010). While the effects of exercise on the immune system are now well established (Walsh et al. 2011a,b) a more recent theme to emerge is whether exercise influences ageing of the immune system, also referred to as immunosenescence.

The purpose of this article is to provide a brief summary of well-established findings in exercise immunology and immunogerontology, before discussing a recent theory that exercise might exert anti-immunosenescence effects on T cells (Simpson 2011; Simpson and Guy 2009). For the concepts that have been covered with limited depth in this article, readers are directed towards some excellent work that has reviewed; the effects of exercise on immune function (Gleeson et al. 2011; Pascoe et al. 2014; Shephard 2003; Walsh et al. 2011a, b); the influence of chronological age and infection history on immunosenescence (Appay and Sauce 2014; Fulop et al. 2013; Pawelec 2012, 2013); and how exercise might slow the rate of immunological ageing or perhaps even rejuvenate aged immune systems (Kohut and Senchina 2004; Muller and Pawelec 2013; Simpson 2011; Simpson and Guy 2009; Simpson et al. 2012). As the majority of studies investigating exercise-associated changes in immune function have focussed on “aerobic” or cardiovascular exercise (e.g., walking, running, cycling), or whether individuals can be classified into distinct, but related categories, of being sedentary, inactive, or physically active, studies focussing on resistance exercise (e.g., weight lifting) will not be discussed. Where relevant, methodological considerations that influence the interpretation of results will be highlighted and areas for future research proposed.

## Associations between the “dose” of exercise, immune function and general health

The influence of exercise on aspects of human immune function has been examined in the short-term, such as in the minutes and hours after a single bout of exercise, after long-term exercise training interventions (e.g., months or occasionally years of regular structured exercise), or with cross-sectional and sometimes longitudinal studies. Generally acute and chronic exercise is thought to be beneficial for immune function, however a U- or J-shaped relationship with infection risk, that is inversely proportional to immune competence, has been proposed (Gleeson and Walsh 2012; Walsh et al. 2011a, b). This “dose” dependent effect is a product of the mode, duration, intensity, and frequency of exercise, which in the context of ageing, culminates in a lifetime exposure and possible accumulation of effects. For example, prolonged periods of sedentary behaviour accumulated over weeks, months or years (i.e., sitting, with an energy expenditure of <1.5 METs—metabolic equivalents; 3.5 mL O_2_ kg^−1^ BM min^−1^) (Sedentary Behaviour Research 2012) is associated with morbidity and mortality from disease, perhaps due to a number of deleterious immunological and inflammatory alterations (Arem et al. 2015; Kanneganti and Dixit 2012; Kraus et al. 2015; Lee et al. 2012; Sedentary Behaviour Research 2012). However, acute bouts of moderate-intensity (3–6 METs or 50–69 % of maximal oxygen consumption; ${\dot{\text{V}}\text{O}}_{{ 2_{ \hbox{max} } }}$) and vigorous-intensity exercise (>6 METs or ≥70 % ${\dot{\text{V}}\text{O}}_{{ 2_{ \hbox{max} } }}$) “stimulate” aspects of immune function (Walsh et al. 2011a, b). Moreover, if this form of exercise is undertaken regularly, so that age-specific guidelines concerning the recommended duration and frequency of exercise are met (e.g., 30 min of moderate intensity exercise on 5 days of the week for adults), the result is a better functioning immune system and a lower incidence of disease (Arem et al. 2015; Lee et al. 2012; World-Health-Organisation 2010). Conversely, some evidence shows that long-duration one-off bouts of exercise (e.g., marathon running, especially if this is unaccustomed) and weeks or months of very high-intensity long-duration athletic training, sometimes referred to as “overreaching” or “over-training”, are associated with an increased risk of certain infections, suggesting compromised immunity (Bonini et al. 2015; Derman et al. 2014; Gleeson and Walsh 2012; Meeusen et al. 2013). At the far end of this scale, it is known that extreme exercise, such as ultra-endurance races (i.e., exercise performed over large parts of a day or several consecutive days with little or no rest), is associated with very large, and sometimes prolonged, immunological and inflammatory disturbances (Turner et al. 2011, 2013). If repeated frequently over a lifetime, it has been suggested that this form of exercise might be associated with an increased risk of cardiovascular disease, and possibly impaired immune function, that could exacerbate ageing processes (Knez et al. 2006; Turner et al. 2014a).

Although the summary above represents current thinking in exercise immunology, the interpretation of results from individual studies is dependent on critical methodological considerations. For example, acute exercise-induced changes in some immunological variables (e.g., blood lymphocyte number, phenotype and cell subset composition) can only be characterised accurately if sample timing is optimal due to transient lymphocyte trafficking patterns (for a time course of changes, see Gabriel and Kindermann 1997; Gleeson and Bishop 2005). Further, measurements of lymphocyte function for example, are strongly influenced by the numbers and proportions of cell types comprising the total lymphocyte pool at the time of sampling, highlighting the need for measurements to be made on a single-cell or single-phenotype basis (Lavoy et al. 2013). With chronic studies, measurements should be made 36–48 h after a final bout of exercise to avoid capturing changes induced by acute exercise (Thompson et al. 2010). Finally, in cross-sectional and longitudinal studies, a number of methods have been used to determine whether participants engage in exercise, ranging from self-report questionnaires and diaries, to direct measurement of ${\dot{\text{V}}\text{O}}_{{ 2_{ \hbox{max} } }}$ with indirect calorimetry, or use of accelerometer-based monitors to objectively quantify the duration and intensity of exercise undertaken. Although using precise and sensitive activity monitors might seem robust, there are a number of considerations when interpreting this information (Thompson and Batterham 2013; Thompson et al. 2009). For example, rather than classifying individuals as being “active” or “inactive”, there is now a greater emphasis on creating multidimensional physical activity profiles that reflect an individual’s engagement in all behaviours (i.e., low, moderate and vigorous activity balanced by time spent being sedentary) (Thompson et al. 2015). This is important because individuals can accumulate substantial sedentary time outside of regular structured exercise (Sedentary Behaviour Research 2012) and therefore counter-intuitively, can be classified as being *both* “active” and “sedentary” at the same time. Even defining individuals as being “active” (i.e., meeting physical activity recommendations) is open to interpretation. For example, depending on the specific criteria used from physical activity guidelines, and the analytical decisions made, it is possible to describe the same individual as “active” but also “inactive” (i.e., failing to meet physical activity recommendations) (Thompson et al. 2009). These methodological considerations demonstrate that the results of studies must be interpreted with caution.

## Immune function and ageing: can exercise help?

Ageing results in alterations to almost all aspects of innate and adaptive immune function, and these changes are typically assumed to reflect deregulation. Alterations to innate immunity include impaired phagocytosis and chemotaxis of neutrophils and monocytes/macrophages, with the latter exhibiting defective antigen processing and presentation, and an inflammatory phenotype (Panda et al. 2009). Ageing is also associated with a lower number, and altered tissue distribution of dendritic cells, which exhibit impaired antigen processing and presentation, along with decreased co-stimulatory and migratory capacity (Panda et al. 2009). Although the number of natural killer (NK) cells increases with age, cytokine production and cytotoxicity on a per cell basis decreases (Panda et al. 2009). Age-associated alterations in adaptive immunity are thought in part, to be driven by an ageing haematopoietic stem cell niche and fewer circulating stem cells, which in turn, are characterised by intrinsic damage and a phenotype skewed towards the myeloid lineage (Geiger et al. 2013). These characteristics, in combination with thymic involution, result in decreased numbers and proportions of CD4+ and CD8+ naïve T cells (Appay and Sauce 2014; Fulop et al. 2013; Pawelec 2012, 2013). Late-stage differentiated effector memory CD4+ and CD8+ T cells accumulate with ageing and many are specific for latent Herpes viruses, especially *Cytomegalovirus* (CMV) (van Lier et al. 2003). Studies examining CD4+ T-helper (Th) cells in the context of ageing, have traditionally shown, based on their signature cytokine profiles, that cells exhibiting a Th2 profile (i.e., IL-4 producing cells) dominate over cells with a Th1 profile (i.e., IFN-γ producing cells) in the elderly (Deng et al. 2004; Shearer 1997). However, more recent focus has been on a possible accumulation of Th17 cells with age (i.e., IL-17 producing cells that are associated with autoimmunity and inflammatory disease) (Schmitt et al. 2013). In addition, attention has also turned to natural- and inducible- regulatory T cells (nTREG and iTREG respectively) and it has been suggested that the number of nTREGs increase with ageing whereas iTREGs decrease (Fessler et al. 2013; Jagger et al. 2014). It is unclear however, if the suppressive capability of these cells is altered with ageing, so the implications of a change in cell numbers are unknown (e.g., high suppressive activity could confer a greater risk of cancer, whereas impaired suppressive activity could confer a greater risk of autoimmune disease). As with T cells, ageing is associated with a decline in naïve B cell production, an accumulation of memory B cells with limited specificities, and less robust plasma cell responses to antigen, with the antibodies produced being less effective (Siegrist and Aspinall 2009).

Importantly, acute bouts of exercise have been shown to improve or “stimulate” aspects of immune function that decline with ageing (Walsh et al. 2011b). Cross-sectional studies have highlighted that “active” individuals exhibit better immune function than “inactive” individuals, even in the elderly (Kohut and Senchina 2004; Simpson et al. 2012). Some support is provided by studies examining chronic exercise training interventions. Data from these studies in the young and elderly show an anti-inflammatory effect of exercise, but long-term changes to immune cell function and phenotype are less consistent (Gjevestad et al. 2015; Gleeson et al. 2011; Kohut and Senchina 2004; Simpson et al. 2012). The acute effects of exercise include improvements in the trafficking and functional capabilities of many cell types, including haematopoietic stem cells (Thijssen et al. 2006), cells of myeloid lineage (e.g., monocytes, neutrophils, dendritic cells) and cells of lymphoid lineage (e.g., NK cells, B cells and T cells) (Shephard 2003; Walsh et al. 2011b). Moreover, acute bouts of exercise (and also exercise training interventions) have been shown to improve in vivo immune responses in the young and elderly, such as antibody responses to influenza vaccines (Pascoe et al. 2014). It is beyond the scope of this article to discuss these effects in detail, but the reader is directed to several excellent articles that have covered this information in depth (see introduction and overview).

Although many aspects of immune function change with ageing, and some of these alterations can be restored transiently by exercise, very few immune parameters are predictive of mortality and/or accepted as “hallmarks” of an ageing immune system (see next section) (Pawelec 2012, 2013). It remains unclear whether exercise can prevent or reverse changes in most of these established hallmarks, however a recent theory has proposed that exercise might elicit preventative and/or restorative effects on T cell immunosenescence (Simpson 2011; Simpson and Guy 2009). The next section will outline “accepted” biomarkers of human immunological ageing, before describing the theory that exercise might invoke an anti-immunosenescence effect on T cells.

## Established hallmarks of immunosenescence and the immune risk profile

The search for robust biomarkers of an ageing immune system has been undertaken for many years. Early studies indicated that the magnitude of T cell proliferative responses to mitogens in vitro is positively associated with survival in the elderly (Roberts-Thomson et al. 1974; Wayne et al. 1990). In addition, higher inflammatory activity, measured by plasma or serum interleukin (IL)-6, tumour necrosis factor-α (TNF-α) and C-reactive protein (CRP), has also been associated with shorter survival (Baune et al. 2011; Bruunsgaard et al. 2003; Giovannini et al. 2011). While innate inflammatory processes dubbed “inflammaging” have traditionally been a focus of research (Franceschi et al. 2000b, 2007), more recent emphasis has been placed on the adaptive immune system. For example, accepted hallmarks of immunological ageing are low numbers and proportions of naïve T cells (in particular CD8+ T cells) and high numbers of memory T cells (especially late-stage differentiated CD8+ T cells) (Appay and Sauce 2014; Fulop et al. 2013; Pawelec 2012, 2013). Longitudinal studies of octogenarians in exceptional health, and nonagenarians, with health profiles more representative of this age group, revealed a cluster of parameters that were referred to as the “Immune Risk Profile” (IRP) (Pawelec et al. 2001; Wikby et al. 1994, 1998). These parameters included low numbers and proportions of B cells, high numbers and proportions of late-stage differentiated CD8+ T cells (i.e., CD27 − CD28−), poor T cell proliferative responses to mitogens, and a CD4:CD8 ratio of <1.0 (Wikby et al. 1994, 1998). In addition, infection with CMV was also clustered into the IRP (Olsson et al. 2000) and together, these characteristics predicted greater all-cause mortality at 2-, 4- and 6-year follow up. Later work showed that IL-6 was also associated with frailty, cognitive decline and mortality (Wikby et al. 2005, 2006). Indeed many features of an ageing immune system appear to be driven by CMV, and infection with this virus alone has since been associated with frailty (Haeseker et al. 2013), cognitive impairment (Gow et al. 2013), poor responses to vaccination in young and elderly individuals (Derhovanessian et al. 2012; Frasca et al. 2015; Moro-Garcia et al. 2012; Turner et al. 2014b; Wald et al. 2013), and ultimately earlier mortality (Savva et al. 2013; Simanek et al. 2011; Spyridopoulos et al. 2015).

A direct and causal role for CMV in ageing processes and disease continues to be investigated and some relationships remain contentious. For example, some studies have shown no effect of CMV on vaccine responses in the elderly (den Elzen et al. 2011; O’Connor et al. 2014; Wald et al. 2013) and other studies have even reported vaccine-boosting effects of CMV in young individuals (Furman et al. 2015). Despite inconsistent results over the effects of CMV on antibody responses to vaccination, studies consistently show that elderly individuals, exhibiting other established hallmarks of an ageing immune system, respond poorly to vaccines (Haq and McElhaney 2014; Strindhall et al. 2016). Encouragingly though, even in vaccine studies that have not directly investigated, characterised or controlled for the degree of immunological ageing in the volunteers examined, it has been shown that acute and chronic exercise can improve the response to this in vivo novel antigen challenge (de Araujo et al. 2015; Pascoe et al. 2014). Thus, a key question that remains is whether exercise exerts an anti-immunosenescence effect, perhaps delaying the onset of immunological ageing or even rejuvenating aged immune profiles.

## Does exercise make immunological “space”? A proposed anti-immunosenescence effect of exercise

It has been proposed that regular bouts of exercise might prevent overcrowding of immunological “space” by expediting the apoptosis of senescent and functionally exhausted late-stage differentiated T cells, many of which are specific for CMV (Simpson 2011; Simpson and Guy 2009). It is proposed that three processes bring about this effect. First, as shown by many investigations (e.g., Campbell et al. 2009; Simpson et al. 2007, 2010; Turner et al. 2010), cells of a late-stage differentiated phenotype are mobilised into peripheral blood during exercise. Second, in line with current thinking in exercise immunology (Dhabhar 2014; Walsh et al. 2011a, b), these cells extravasate from blood, homing to peripheral and/or inflamed tissues 1–2 h after exercise. Here, it is proposed that cells are exposed to a number of pro-apoptotic stimuli (e.g., reactive oxygen species glucocorticoids, cytokines) that cause apoptosis. This assertion is partly supported by observations in mice, showing that lymphocyte apoptosis occurs post-exercise in tissues that are thought to be the homing destination of mobilised cells (Kruger et al. 2009). Third—the final stage of this hypothesis—it is proposed that the naïve T cell repertoire is able to expand in response to the immunological “space” that has been created, initiated by a hypothetical negative feedback loop that governs the number of naïve and memory cells. Further, it is suggested that the production of naïve cells is brought about by exercise-induced thymopoiesis and/or extrathymic T cell development in tissues such as the liver. A post-exercise surge in thymopoiesis is possible considering that skeletal muscle secretes IL-7 (Haugen et al. 2010) which might in turn increase thymic mass and lead to the emergence of recent thymic emigrants (Fry and Mackall 2005). While some parts of the “exercise makes immunological space” hypothesis are supported by classical findings in the exercise immunology literature, this theory is also dependent on several themes that continue to be debated in mainstream immunology. These concepts and recent experimental evidence supporting or refuting the framework presented by Simpson and Guy (2009) and Simpson (2011) are discussed below with suggestions for how future research could robustly test the ideas proposed.

## Are late-stage differentiated T cells senescent?

Infection with CMV causes a large accumulation of virus-specific memory T cells, so that approximately 10 % of the CD4+ and CD8+ T cell pool becomes specific for this virus (Sylwester et al. 2005). Large inter-individual differences exist, and it has been reported that 23 % of the CD8+ T cell pool can become specific for just a single CMV epitope (Khan et al. 2002). These CMV-specific T cells exhibit a late-stage differentiated phenotype, including loss of co-stimulatory molecules (e.g., CD27 and CD28) and cell-surface proteins associated with homing to lymphoid tissue (e.g., CD62L and CCR7) (Appay et al. 2008; van Lier et al. 2003). Further, these cells re-express CD45RA in combination with markers often stated to reflect “senescence” such as CD57 and KLRG1 (Appay et al. 2008; van Lier et al. 2003). Classifying these cells as “senescent” however is subject to debate, and is dependent on what constitutes the definition of senescence (e.g., loss or expression of key cell-surface molecules, functional capacity, such as the ability to proliferate, produce cytokines and kill targets, or telomere length) (Koch et al. 2007; Pawelec 2013).

One of the first studies to describe CMV-specific cells as being dysfunctional, did so because the proportion of antigen-stimulated interferon-gamma (IFN-γ) producing cells from elderly people, was lower than in young people (Ouyang et al. 2003a). However, the cumulative IFN-γ production was higher in the elderly due to greater absolute numbers of these cells compared to the young (Ouyang et al. 2003a). Rather than “senescence” per se, this might reflect a mechanism to limit excessive inflammatory activity when controlling CMV in the elderly. The view that many of these late-stage differentiated cells are not dysfunctional or senescent is supported by studies showing that the inhibitory molecule programmed death-1 (PD-1) is expressed on very few CMV-specific cells and at low levels (Petrovas et al. 2006; Trautmann et al. 2006). Moreover, recent work has shown that many CMV-specific CD8+ T cells, identified using MHC-class I tetramers, are multifunctional, producing IFN-γ, IL-2 and TNF-α (Riddell et al. 2015). Therefore these cells might not classify as being senescent due to this multifunctional phenotype alone. In addition, these cells have telomeres of intermediate length, despite “senescent” cell-surface characteristics (e.g., CD45RA + CD27−) (Riddell et al. 2015). Thus, despite being capable of cytokine production and exhibiting potent cytotoxic activity, if these cells fail to *proliferate* in response to antigen (i.e., a key characteristic of senescence) then this is due to mechanisms other than replicative senescence, such as activation of p38 mitogen-activated protein kinase signalling by reactive oxygen species (Henson et al. 2014). Indeed, cells failing to proliferate can exhibit a profile that is distinct from replicative senescence, referred to as stress-induced premature senescence (SIPS) (Toussaint et al. 2002a, b). This state, brought about by exposure to sub-cytotoxic stress (e.g., by reactive oxygen species) is characterised by growth arrest and adoption of a senescence-associated secretory phenotype, whereby inflammatory cytokines and transforming growth factor-beta (TGF-β) are produced (Toussaint et al. 2002a, b). Studies have shown that cells in a state of SIPS, brought about independently of telomere shortening, exhibit a proteomic profile that is distinct from cells that have undergone replicative senescence (Aan et al. 2013; Dierick et al. 2002).

To conclude, for the reasons described above, it is imprecise to refer to all late-stage differentiated T cells as being “senescent”. Analysis of cell surface proteins alone is not sufficient to discriminate between fully functioning late-stage differentiated cells and those that are truly senescent and functionally exhausted (see next section). It is also important to examine whether cells have become senescent due to sub-cytotoxic stress (e.g., from reactive oxygen species exposure) or due to telomere shortening. In relation to the “exercise makes immunological space” hypothesis, it might be questioned whether it is advantageous to remove some late-stage differentiated T cells in the first place, or at least, whether it is possible to eliminate only the cells that are functionally exhausted.

## Is it advantageous to *remove* senescent late-stage differentiated T cells, or should interventions focus on *restoring* cell function?

From an evolutionary perspective, there may be an advantage to having an accumulation of late-stage differentiated T cells, assuming few are senescent/functionally exhausted, and considering that many will be specific for viruses such as CMV that require constant immune-surveillance (van Lier et al. 2003). In the context of solid organ or stem cell transplant, there is substantial morbidity and mortality from CMV reactivation and subsequent disease, in part, due to loss or suppression, of cell-mediated immunity (Stocchi et al. 1999). This finding highlights the important role that fully functioning late-stage differentiated T cells have in controlling latent viruses. The importance of T cells targeting CMV is emphasised by protocols that have been developed to adoptively transfer CMV-specific CD8+ T cells to prevent viral reactivation in transplant recipients (Cobbold et al. 2005). Even in healthy individuals, the latest evidence points towards an essential role for immune-surveillance of CMV. For example, it has been shown that a lower proportion of naïve CD8+ T cells, a higher proportion of memory CD8+ T cells, and a robust pro-inflammatory response to CMV, are associated with better survival in a longitudinal analysis of elderly individuals (Derhovanessian et al. 2013).

To clarify, the “exercise makes immunological space” hypothesis proposes that rather than eliminating late-stage differentiated T cells non-specifically, exercise results in a selective removal of senescent and functionally exhausted cells. However, a key question that remains is what proportion of the T cell pool needs to be specific for CMV to ensure adequate viral control (reviewed in Borchers et al. 2014). It is therefore currently unknown whether values such as 10 % of the CD4+ and CD8+ T cell pool being specific for CMV is above, or below, the ideal threshold (Sylwester et al. 2005). Studies can estimate the extent of CMV “obsession” by identifying antigen-specific cells using peptide-MHC class-I or class-II multimers (Altman et al. 1996; Davis et al. 2011). However, this methodology is limited to common human leukocyte antigen (HLA)-types and is usually restricted to immunodominant antigens rather than the full spectrum of virus proteins that T cells target (Borchers et al. 2014). Thus, it is very difficult to enumerate all CMV-specific cells in an individual using this technology. Moreover, this methodology will not identify the proportion of antigen-specific cells that are senescent/exhausted, unless functional measurements are incorporated in tandem or post hoc (Borchers et al. 2014; Klenerman et al. 2002). Studies using other approaches, such as cytokine measurement in response peptide stimulation, will only detect functional cells, providing an underestimation of the total number of CMV-specific cells, and failing to quantify those that are senescent/exhausted (Borchers et al. 2014; Klenerman et al. 2002). Finally, it is possible that the threshold for protective CMV-specific immunity will be different between immune-competent individuals, those exhibiting an IRP-like phenotype, and immune-compromised patients (Borchers et al. 2014; Gamadia et al. 2001; Ouyang et al. 2003b).

Even if an ideal threshold for the number of functional CMV-specific T cells is defined, and if it can be proven that exercise is capable of selectively removing senescent/exhausted cells, an alternative anti-immunosenescence strategy might be to intervene in ways that *restore* cell function. For example, during active and chronic infection with CMV, the small number of dysfunctional T cells specific for this virus express high levels of PD-1 and exhibit impaired IL-2 production and proliferation (Antoine et al. 2012; Dirks et al. 2013). Studies have shown that these functional impairments can be restored by PD-1 blockade (Antoine et al. 2012; Dirks et al. 2013). However, due to financial costs and risk of side effects, immune checkpoint inhibitors will likely be limited to treating certain cancers, where anti-PD1 therapies are being used to target anergic tumour-specific T cells (Naidoo et al. 2014).

Rather than restoring aspects of cell function with immunotherapies that come with considerable cost and risk, reducing T cell exposure to reactive oxygen species might be a more preferable feature of anti-immunosenescence interventions. This idea is justified because inhibition of the p38 mitogen-activated protein kinase signalling pathway, which if activated by excess reactive oxygen species prevents proliferation, restores the proliferative capacity of cells exhibiting “senescent” phenotypes (Henson et al. 2014). In support, an anti-immunosenescence effect of the antioxidant compounds Ebselen and *N*-acetyl-cysteine on CD4+ T cell clone cultures from healthy young and middle-aged donors has been shown (Marthandan et al. 2013). Supplementing cultures with these antioxidants substantially increased the lifespan and proliferative capacity of T cell clones, which occurred in parallel with an increase in the ratio of reduced glutathione (GSH) to oxidised glutathione (GSSG) and lower oxidative DNA damage (Marthandan et al. 2013). Critically, these effects were not replicated in T cell clones from an elderly donor (Marthandan et al. 2014) despite conforming to SENIEUR protocol criteria (Ligthart et al. 1984, 1990), suggesting there may be an age threshold before which anti-immunosenescence interventions must be initiated. The beneficial effects of antioxidants on aspects of immune function are not limited to healthy individuals and in vitro experiments. For example, one study has shown that high dose vitamin E supplementation (in combination with selenium and vitamin C administered in line with recommended dietary allowance values) is associated with an improved capacity of T cells to produce IL-2 and IFN-γ, along with an increased CD4:CD8 ratio in advanced colorectal cancer patients (Malmberg et al. 2002). More broadly, dietary supplementation with selenium has been shown to reduce the risk of developing several diseases with immune or inflammatory aetiologies, delay disease progression in patients with these conditions, and improve in vivo immune responses via a number of immunological mechanisms (Huang et al. 2012; Rayman 2012; Steinbrenner et al. 2015). Thus, rejuvenating rather than removing senescent late-stage differentiated T cells, by for example manipulating diet, seems to be a feasible intervention that could have anti-immunosenescence effects.

## Are senescent late-stage differentiated T cells susceptible to apoptosis?

If it were to be accepted that senescent T cells should be removed as part of an anti-immunosenescence strategy, then it must be considered how this process can be achieved. For example, it has been proposed that CMV-specific T cells could be physically removed (e.g., repeated blood samples and/or T cell targeting antibody therapy) and replaced by adoptive transfer of naïve T cells (Lang et al. 2012). This invasive and high-risk strategy is unlikely to gain popularity in an ageing but otherwise healthy population. Thus, an exercise-based anti-immunosenescence intervention is appealing. In the “exercise makes immunological space” hypothesis it has been proposed that late-stage differentiated T cells travel to tissues and the senescent and functionally exhausted sub-population are eliminated by apoptosis (Simpson 2011; Simpson and Guy 2009). However, on the basis of in vitro experiments, it is commonly cited that “senescent” CD8+ T cells are resistant to apoptosis (Spaulding et al. 1999). In addition, CMV-specific CD8+ T cells measured in peripheral blood have been shown to exhibit high levels of the anti-apoptotic protein Bcl-2 (Khan et al. 2002), which might render them insensitive to cell death induced the Fas-receptor (Fas-R) interacting with Fas-ligand (Fas-L). For these reasons, resistance to apoptosis (e.g., due to replicative senescence or SIPS) has been put forward as one explanation for why these cells accumulate over a lifetime (Franceschi et al. 2000a; Pawelec et al. 2004). It appears however, that CD4+ T cells become *more* susceptible to apoptosis with in vitro age (Pawelec et al. 1996). This might explain the low CD4:CD8 ratios exhibited in some CMV-infected elderly individuals, and the less dramatic effects of CMV on the CD4+ T cell repertoire (Fulop et al. 2013; Pawelec 2013).

In light of this discussion, it remains unclear whether senescent and functionally exhausted late-stage differentiated T cells can be eliminated by apoptosis in the tissues following exercise. Even if this process does occur, research must also identify the signals triggering apoptosis, to indicate which of the two apoptotic pathways is responsible. For example, the extrinsic apoptotic pathway, characterised by a ligand binding to its receptor (e.g., Fas-L to Fas-R, or TNF-α to TNF-receptor 1), results in the production of a death-inducing signalling complex that activates caspases which degrade intracellular targets and induce cell death (Kruger and Mooren 2014; Mooren and Kruger 2015b). On the other hand, the intrinsic pathway, characterised by non-receptor mediated stimuli such as intracellular stress (e.g., high reactive oxygen species), results in permeabilization of the outer mitochondrial membrane, release of cytochrome-c, and activation of several caspases ultimately inducing cell death (Kruger and Mooren 2014; Mooren and Kruger 2015b). In the “exercise makes immunological space” hypothesis, potential apoptotic triggers are highlighted as being reactive oxygen species and glucocorticoids, that would likely trigger the intrinsic pathway, but also Fas-L/Fas-R interaction or cytokines, that would likely trigger the extrinsic pathway (Simpson 2011; Simpson and Guy 2009). Considering the large changes in redox balance induced by exercise (Finaud et al. 2006), reactive oxygen species might be the most relevant apoptotic trigger post-exercise. However, the extrinsic apoptotic pathway should not be ruled out considering that Fas-dependent signalling has been implicated in post-exercise lymphocyte apoptosis in the tissues (Kruger et al. 2009).

Further understanding would be gained by examining apoptosis of phenotypically characterised (and ideally virus-specific) T cells in response to the apoptotic signals most likely to be encountered in the tissues post-exercise. In support of the framework proposed by Simpson and Guy (2009) and Simpson (2011) T cells expressing cell-surface proteins associated with differentiation or “senescence” (e.g., CD57 and KLRG1) are more susceptible to H_2_O_2_ induced apoptosis than total lymphocytes (Wang and Lin 2010) and cells of a naïve phenotype (e.g., CD45RA + CCR7+) in vitro (Takahashi et al. 2005). However, studies examining virus specificity and apoptosis have provided mixed results. For example, further support is provided by work that has shown CMV-specific CD8+ T cells, rather than being resistant to apoptosis, are equally as susceptible to Fas-L/Fas-R induced apoptosis as the total pool of CD8+ T cells (Mueller et al. 2001). These results, when interpreted with other data showing that CMV-specific cells exhibit high levels of the anti-apoptotic protein Bcl-2 (Khan et al. 2002), emphasise that programmed cell death is a balance between pro- and anti-apoptotic signals. However, perhaps in opposition to the framework by Simpson and Guy (2009) and Simpson (2011), work in the context of Epstein Barr Virus (EBV) infection, has shown that CD45RA re-expressing EBV-specific CD8+ T cells are resistant to growth factor deprivation-induced apoptosis, and this was associated with greater expression of Bcl-2 (Dunne et al. 2002). Thus, it is yet to be determined whether CMV-specific late-stage differentiated T cells are susceptible to possible apoptotic signals that might be encountered in the tissues post-exercise.

## Is the size of immunological “space” fixed?

Historically, it has been thought that thymic output of naïve T cells is negligible after adolescence, which in part, led to the view that there might be an upper limit to the number of T cells in the immune system (Franceschi et al. 2000a). This implies there is a fixed number of naïve T cells capable of mounting responses to novel antigens encountered over a lifetime (Franceschi et al. 2000a). Thus, naïve T cells might eventually be “used up” due to on-going differentiation into memory cells that “fill up” immunological “space” (Franceschi et al. 2000a). Further, it has been suggested that the massive accumulation of CMV-specific T cells upon infection may lead to a “squeezing out” of T cells targeting less dominant viruses (e.g., Varicella Zoster Virus; VZV) or non-persistent infections such as Influenza (Akbar and Fletcher 2005). In the context of VZV, it is possible that this process might underlie loss of viral control, subsequent reactivation, and manifestation of shingles later in life (Berger et al. 1981). While studies continue to support the age-associated reduction in thymic function, evidence now points towards a more gradual decline, whereby thymic output persists, albeit reduced, up until around 70 years of age (Douek et al. 1998; Ferrando-Martinez et al. 2010).

Although a decline in thymic output with ageing is accepted, the concept of a fixed amount of immunological space has been debated (Huster et al. 2009; Schwanninger et al. 2007; van Leeuwen et al. 2006; Vezys et al. 2009). For example, in an analysis of total CD8+ T cells and virus-specific CD8+ T cells from CMV seronegative patients before and after transplantation with a CMV-infected kidney, it was concluded that there is a long-lasting enlargement of the CD8+ T cell compartment (van Leeuwen et al. 2006). By analysing the numbers, proportions and phenotype of cells, an appearance of CMV-specific T cells was shown following kidney transplant, and these cells expanded substantially, acquiring differentiated profiles. However, the absolute number of pre-existing EBV-specific and Influenza-specific T cells remained stable (van Leeuwen et al. 2006). Although other studies have shown a decline in pre-existing memory cells with ageing, perhaps an artefact of measuring proportions rather than the absolute number of cells, examining a potential decline in virus-specific T cell *function* might be more important and relevant (Huster et al. 2009; Khan et al. 2004).

It is yet to be agreed if the size of the T cell pool is fixed. Encouragingly, it is also not certain if such a “space” constraint would limit the potential for exercise to invoke an increase in naïve T cells, nor whether the number of these cells is regulated by negative feedback. Indeed, perhaps a more important goal of exercise, rather than removing expanded clones of senescent and functionally exhausted T cells, might be stimulation of the thymus, subsequently increasing naïve T cell output (Appay and Sauce 2014; Goronzy et al. 2015). It is unknown if thymic output is dependent upon the amount of available “space”, but it is possible these are independent processes. Further, an increase in naïve T cells might not necessarily be thymically derived—a phenomenon that has been observed in young adults thymectomised during childhood (Torfadottir et al. 2006). Compared to T cells that mature in the thymus, of which most express an αβ T cell receptor (TCR) and few express a γδ TCR, a higher proportion of extra-thymically matured cells express a γδ TCR and many of these cells, rather than expressing an α and β chain of the CD8 molecule, express a CD8 αα homodimer (Torfadottir et al. 2006). Analysis of T cell phenotype on this basis might help answer the question of whether a possible exercise-induced increase in naïve T cells is thymically or extra-thymically derived.

Finally, an exercise-associated replenishment of naïve T cells might be elicited earlier in the T cell development process. For example, a recent murine study has shown that 24 h after an acute bout of exercise, lymphocytes undergo apoptosis in peripheral blood and bone marrow, which occurs in parallel with an increase of haematopoietic stem cells at these sites (Mooren and Kruger 2015a). Subsequent experiments showed that after inducing apoptosis in splenic lymphocytes from resting mice by H_2_O_2_ exposure, and injecting apoptotic cells (and apoptotic supernatant separately), into the bloodstream of other resting mice, there was a substantial mobilisation of haematopoietic stem cells 2 h later (Mooren and Kruger 2015a). These mobilised haematopoietic stem cells might travel to the thymus (or potentially extra-thymic sites) and develop into naïve T cells (Radtke et al. 2013).

While it is appealing for an anti-immunosenescence effect of exercise to bring about changes in the T cell repertoire, this may not be essential, and instead, other aspects of an ageing immune system could be the targets of exercise. For example, the necessity for an on-going supply of naïve T cells has only been justified at a conceptual level, whereby it has been assumed the decline in naïve cells increases the susceptibility to infection (Franceschi et al. 2000a). This idea has not been tested experimentally (Pawelec 2012), therefore anti-immunosenescence effects of exercise may have their roots in other aspects of immune function or might even be brought about indirectly (see next section).

## Could exercise limit the expansion of late-stage differentiated T cells indirectly?

Assuming exercise invokes anti-immunosenescence effects, in part by limiting the expansion of CMV-specific late-stage differentiated T cells, it could be that these changes are brought about indirectly, as opposed to the mechanisms (i.e., deletion and replacement of cells) proposed in the “exercise makes immunological space” hypothesis. Possible indirect effects of exercise, such as limiting viral reactivation, are discussed in this section with reference to overweight and obese individuals—a population likely to be inactive and sedentary who also appear to exhibit signs of immunosenescence that could be countered indirectly by exercise.

Overweight or obese individuals exhibit a greater incidence of diseases characterised by immunological and inflammatory pathology, for which some of the underlying mechanisms derive from adipose tissue accumulation and deregulation (Kanneganti and Dixit 2012; Tchernof and Despres 2013). Other more subtle immunological observations, hinting at a state of early or exacerbated immunosenescence, have also been made in the context of obesity. For example, obese individuals are at a greater risk of viral and bacterial infections, have longer stays in hospital and exhibit more frequent and prolonged complications, such as antibiotic treatment failure (Falagas and Kompoti 2006; Gottschlich et al. 1993; Karlsson et al. 2012; Longo et al. 2013). Further, obese individuals exhibit poor antibody responses to vaccination (Clemens et al. 1997; Eliakim et al. 2006; Karlsson et al. 2012; Sheridan et al. 2012; Talbot et al. 2012), impaired lymphocyte proliferation to mitogens (Nieman et al. 1999), and there is an inverse correlation between body mass index (BMI) and leukocyte telomere length measured in peripheral blood (Muezzinler et al. 2014). Although limited, there is some evidence that the T cell pool in obese individuals has immunosenescent characteristics too. For example, one report has shown that the T cell pool is skewed towards a T-regulatory and Th2 phenotype that is also seen with ageing (van der Weerd et al. 2012). Another report has shown that with obesity there is an accumulation of terminally differentiated effector memory γδ T cells with impaired anti-viral function (Costanzo et al. 2015). Finally, there is at least some indication that with obesity, αβ T cells exhibit the classic phenotypes associated with immunosenescence, even in children (Alam et al. 2012; Spielmann et al. 2014).

If obesity is associated with immunosenescence, then it is likely that some of the observed immunological alterations are driven by repeated antigenic stimulation with CMV as with non-obese populations (Appay and Sauce 2014; Fulop et al. 2013; Pawelec 2012, 2013). However, CMV reactivation might be even more frequent in obesity, because excess adiposity drives chronic systemic inflammation and oxidative stress (Aroor and DeMarco 2014; Cervellati et al. 2014; de Heredia et al. 2012; Kanneganti and Dixit 2012; Le Lay and Simard 2014), which in turn can reactivate CMV directly (Cinatl et al. 1999; Docke et al. 1994; Jaganjac et al. 2010; Prosch et al. 1995; Speir 2000; Stein et al. 1993; Vossen et al. 1997). Thus, considering exercise decreases visceral and subcutaneous adiposity even in the absence of “weight loss” (Ross and Janiszewski 2008; Tchernof and Despres 2013), and exercise is a potent anti-inflammatory stimulus that helps maintain redox balance even in obesity (Farinha et al. 2015; Gleeson et al. 2011; Ji et al. 2006; Radak et al. 2008), then exercise might reduce viral reactivation in lean, overweight and obese individuals through these mechanisms, limiting the accumulation of late-stage differentiated CMV-specific T cells.

The next section will summarise the latest evidence supporting or refuting the theory that “exercise makes immunological space” which proposes that exercise might prevent, delay or even restore immunosenescent profiles, by mechanisms that directly target senescent and functionally exhausted T cells.

## Does experimental evidence support a role for exercise influencing T cell immunosenescence?

Several recent articles have improved our understanding of whether exercise imparts an anti-immunosenescence effect on T cells. Four studies have compared characteristics of the T cell pool from individuals who differ by cardiorespiratory fitness level or their engagement in different volumes of exercise (Brown et al. 2014; Moro-Garcia et al. 2014; Prieto-Hinojosa et al. 2014; Spielmann et al. 2011). Extending these cross-sectional investigations, two studies have undertaken a repeated and longitudinal analysis of T cells from endurance athletes over a period of approximately six months (Cosgrove et al. 2012; Teixeira et al. 2014).

One of the first papers leading to, and providing experimental evidence for the hypothesis that “exercise makes immunological space”, showed that the percentage of the CD4+ and CD8+ T cell pool comprising late-stage differentiated T cells was inversely correlated with cardiorespiratory fitness (i.e., ${\dot{\text{V}}\text{O}}_{{ 2_{ \hbox{max} } }}$) in 102 males aged 18–61 years (Spielmann et al. 2011). ${\dot{\text{V}}\text{O}}_{{ 2_{ \hbox{max} } }}$ was estimated by measuring ${\dot{\text{V}}\text{O}}_{2}$ at several sub-maximal exercise intensities, with extrapolation to estimate ${\dot{\text{V}}\text{O}}_{2}$ at a predicted maximal workload (Spielmann et al. 2011). Fascinatingly, the age-associated decrease in the proportion of naïve T cells and increase in the proportion of late-stage differentiated T cells, did not withstand statistical adjustment for cardiorespiratory fitness, suggesting that ageing may be secondary to fitness in shaping the T cell repertoire. Most associations withstood adjustments for body composition, and analysis of a sub-group (only approximately one-third of participants) showed that CMV infection, while exerting typical effects on the T cell repertoire, was not associated with cardiorespiratory fitness (Spielmann et al. 2011). Thus, it was concluded that fitter individuals exhibit a smaller age-associated decline in naïve T cells and a smaller accumulation of late-stage differentiated T cells.

In line with the idea that “exercise makes immunological space”, if bouts of exercise result in a selective “deletion” of senescent and functionally exhausted late-stage differentiated T cells, then it might be expected for “active” people to have lower numbers and proportions of late-stage differentiated cells compared to “inactive” people. To date, only one study partly supports this contention. Brown et al. (2014) examined the CD4+ and CD8+ T cell pool in 8 males and 8 females (approximately 18 years of age) who were classified as well-trained (soccer players self-reporting 9–12 h of exercise per week), and in 8 males and 8 females classified as untrained (individuals self-reporting 2–3 h of exercise per week). Supporting an anti-immunosenescence effect of exercise, untrained individuals exhibited the highest proportions of CD4+ and CD8+ late-stage differentiated cells, and the lowest proportions of CD8+ naïve cells (Brown et al. 2014). However, the effects of training status were largely influenced by sex: only untrained males exhibited the high proportions of memory cells and low proportions of naïve cells. Untrained females exhibited similar T cell profiles to trained females, suggesting an anti-immunosenescence effect of oestrogen rather than exercise, or even a pro-immunosenescence effect of testosterone as has been shown previously (Furman et al. 2014).

Studies collecting resting blood samples from endurance athletes and from less-active controls (defined using self-reported physical activity level), suggest that very large volumes of exercise elicit a pro-immunosenescence effect. For example, endurance athletes in the third decade of life (n = 27) exhibited a greater proportion of late-stage differentiated CD4+ (but not CD8+) T cells, a lower CD4:CD8 ratio, poor T cell activation in response to anti-CD3 stimulation, and reduced thymic output of CD8+ T cells, as shown by T cell receptor excision circle (TREC) quantification, compared to less-active age-matched controls (n = 30) (Moro-Garcia et al. 2014). Interestingly, most of these effects were not observed when comparing elderly athletes (n = 12) to age-matched less-active controls (n = 26). This finding suggests that a pro-immunosenescence effect of high-volume exercise might only be apparent in younger adults, and this effect is no worse than the immune decline caused by chronological ageing and/or infection history itself. Another study, examining the CD4+ and CD8+ T cell pool, has reported that endurance athletes (age range 18–36 years, n = 19) have lower absolute numbers of naïve, and higher absolute numbers of memory T cells, compared to less active age-matched controls (n = 16) (Prieto-Hinojosa et al. 2014). In addition, thymic output, assessed by TREC concentration in peripheral blood, was substantially lower in endurance athletes compared to age-matched controls. Interestingly, TREC levels in these young endurance athletes were comparable to those typically seen in the sixth decade of life, and the lowest TREC levels were shown in CD4+ compared to CD8+ T cells (Prieto-Hinojosa et al. 2014). A pro-immunosenescence effect of high-volume exercise is supported by two longitudinal studies; the first examining older endurance athletes (age range 39–50 years, n = 10) (Cosgrove et al. 2012), and the second examining younger endurance athletes (age range 16–19 years, n = 13) (Teixeira et al. 2014). These studies show that over approximately 6 months, the numbers and proportions of CD4+ and CD8+ late-stage differentiated T cells accumulate whereas CD4+ and CD8+ naïve cells decrease (Cosgrove et al. 2012; Teixeira et al. 2014).

Several considerations should be made when interpreting the results of studies summarised in this section. For example, cardiorespiratory fitness does not necessarily reflect engagement in exercise over a substantial period of the lifetime. Compared to individuals who may have been very sedentary, or alternatively, extremely active for a number of years, it might be that the individuals examined by Spielmann et al. (2011) already had a small to moderately-sized accumulation of late-stage differentiated cells at the time of investigation (i.e., these individuals may not represent the extremes of the population). It should also be considered that the methodology used in the majority of studies (i.e., Brown et al. 2014; Moro-Garcia et al. 2014; Prieto-Hinojosa et al. 2014; Spielmann et al. 2011) to define individuals was largely self-report questionnaires, and the characteristics (e.g., age, training load, exposure to exercise over a lifetime, stage of the menstrual cycle, CMV serostatus) was not uniform between studies. Also, several studies only examined the proportion of T cells exhibiting a naïve or memory phenotype (Brown et al. 2014; Cosgrove et al. 2012; Moro-Garcia et al. 2014; Spielmann et al. 2011), when absolute numbers are probably a more important and sensitive measure. In addition, although *within* studies, blood samples are usually collected from volunteers at the same time of day, the timing of samples is not always consistent *between* studies. This is important considering that the kinetics, numbers and function of T cells are known to exhibit a diurnal rhythm, the characteristics of which, can be different with increasing age (Dimitrov et al. 2009; Kirsch et al. 2012; Mazzoccoli et al. 2011). To emphasise, even with broader assessments of immune function, such as antibody responses to vaccination, different effects of the vaccine are shown if it is administered in the morning or afternoon (Langlois et al. 1995; Phillips et al. 2008).

**Fig. 1:**
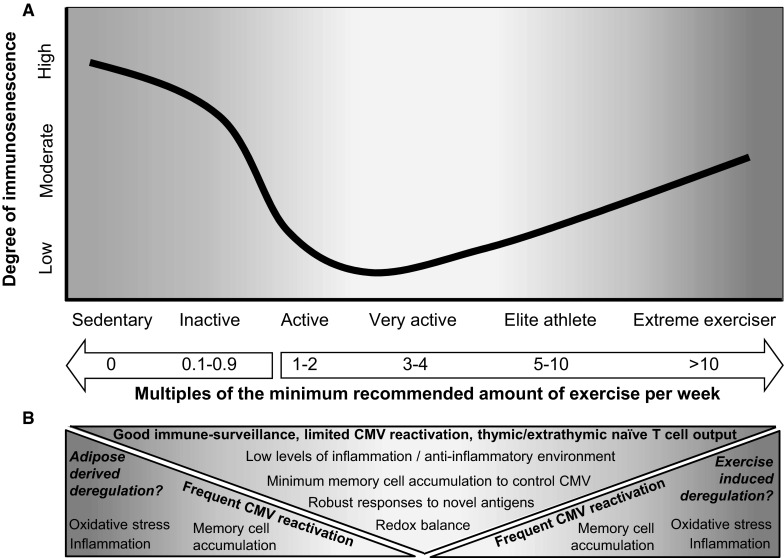
**a** A hypothetical model proposing a possible dose response relationship between exercise and the rate or degree of immunosenesence at a given age. The concept of multiples of minimum recommended amount of exercise per week is modified from Arem et al. (2015). For example, 1 multiple of the minimum recommended level of exercise is 150 min per week of moderate-intensity exercise (i.e., 3–6 METs, 50–69 % maximal oxygen consumption; ${\dot{\text{V}}\text{O}}_{{2_{\hbox{max} } }}$) or 75 min per week of vigorous-intensity exercise (>6 METs or ≥70 % ${\dot{\text{V}}\text{O}}_{{2_{\hbox{max} } }}$) (World-Health-Organisation 2010). Consequently, two multiples represents 300 min per week of moderate-intensity exercise or 150 min per week of vigorous-intensity exercise. The duration of exercise undertaken by elite athletes is based upon typical training volumes for individuals undertaking endurance events (e.g., runners, rowers, cross-country skiers) (Seiler and Tønnessen 2009). Sedentary behaviour includes activities such as sitting with an energy expenditure of <1.5 METs—metabolic equivalents; 3.5 mL O_2_ kg^−1^ BM min^−1^. Sedentary individuals undertake almost no exercise. Inactive individuals take part in a very low volume of exercise, and fail to meet exercise recommendations. Active and very active individuals meet these guidelines, and gain the associated benefits to health and immune function. Exercise recommendations are exceeded by elite athletes and extreme exercisers (the latter include individuals taking part in ultra-endurance training and competition) and this may be associated with immune deregulation. **b** Similar characteristics of immunosenescence are present at each extreme of the exercise-continuum, but the magnitude of effect, and origin of immune deregulation, potentially mediated by inflammation and oxidative stress, is different (e.g., adipose-derived inflammation and oxidative stress in sedentary/inactive individuals, whereas in elite athletes and extreme exercisers, vigorous and prolonged exercise results in excessive inflammatory activity and disturbances to redox balance). It is unclear whether* Cytomegalovirus* (CMV) reactivates because of poor T cell control or due to oxidative stress, inflammation, or excessive adrenergic activity (the latter in elite athletes/extreme exercisers only). Active or very active individuals (middle triangle) exhibit favourable changes to immune function that results in robust and sustained responses to novel antigens. This profile might be induced by the anti-inflammatory effect of regular exercise and maintenance of redox homeostasis. It is unclear whether this model also generalises to CMV seronegative individuals

To summarise, the relationship between the volume of exercise and the rate or degree of immunosenescence is probably not linear, and is likely to be strongly influenced by a lifetime accumulation of exercise behaviour (see Fig. 1). As indicated by work in the context of obesity, individuals who are likely to be classed as both sedentary and inactive, appear to show alterations in the T cell pool indicative of immunosenescence (Alam et al. 2012; Costanzo et al. 2015; Spielmann et al. 2014; van der Weerd et al. 2012). When examining individuals described as being active or those who have been exposed to moderate-to-high volumes of exercise over a short period of the lifespan (e.g., young adults), it is likely that exercise exerts an anti-immunosenescence effect in a dose-dependent manner until a certain threshold (Brown et al. 2014; Spielmann et al. 2011). At the extreme end of the exercise continuum, elite endurance athletes, or ultra-endurance athletes, who have engaged in frequent, intensive and prolonged exercise over a substantial period of time, might exhibit exacerbated immunosenescent profiles compared to active individuals. It is possible this effect is due to latent viral reactivation, mediated by disturbances in redox homeostasis, excessive inflammatory activity or sustained adrenergic activity (Jaganjac et al. 2010; Prösch et al. 2000; Turner et al. 2014a). Thus, based on the limited evidence to date, it appears that the relationship between exercise and T cell immunosenescence is U-shaped. Moreover, it seems that an anti-immunosenescence effect of exercise might only occur up to a threshold of lifetime exercise exposure (e.g., one to three multiples of the duration, intensity and frequency of exercise recommended by physical activity guidelines per week; see Fig. 1).

## Are interventions countering T cell immunosenescence relevant to CMV seronegative individuals?

It might be questioned whether relationships between exercise and T cell immunosenescence generalise to individuals who are not infected with CMV. The “exercise makes immunological space” hypothesis discusses the removal of senescent and functionally exhausted late-stage differentiated T cells. These cells are also referred to as terminally differentiated effector memory cells that re-express CD45RA, or TEMRA cells (Fulop et al. 2013; van Lier et al. 2003). Generally, it is TEMRA cells that become dysfunctional (Fulop et al. 2013; van Lier et al. 2003) and compared to other persistent infections that periodically reactivate (e.g., EBV) it is CMV reactivation that causes an expansion of these cells (Cantisan et al. 2009; Fulop et al. 2013; Gamadia et al. 2004). Thus, as TEMRA cells do not accumulate in elderly CMV seronegative individuals (Wertheimer et al. 2014) a *removal* of senescent T cells probably only applies to those infected with CMV. However, if exercise stimulates thymic or extra-thymic output of naïve T cells, this feature of an anti-immunosenescence intervention might be beneficial for all individuals, considering the age-associated decline of these cells even in those not infected with CMV (Vescovini et al. 2013; Wertheimer et al. 2014).

## Future research to robustly test the anti-immunosenescence effect of exercise

If senescent and functionally exhausted late-stage differentiated T cells are selectively removed following bouts of exercise, then it might be expected for the number of these cells (or the total late-stage differentiated T cell population), measured in resting blood samples, to be lower than pre-exercise values in the hours, days, weeks, months or even years after single or repeated bouts of exercise. However, considering it is known that after exercise the total lymphocyte count returns to pre-exercise levels within approximately 6–10 h (Gabriel and Kindermann 1997; Gleeson and Bishop 2005), then it might be expected for late-stage differentiated cells to follow the same pattern. Initial observations (*n* = 5 healthy well-trained males aged 20–26 years) indicate that the numbers and proportions of late-stage differentiated CD4+ and CD8+ T cells, measured 10 and 24 h after 60 min of treadmill-running at 80 % ${\dot{\text{V}}\text{O}}_{{2_{\hbox{max} } }}$, are not substantially different from pre-exercise levels (Turner et al. 2016). It is important to emphasise however that this work defined late-stage differentiated cells on the basis of CD27, CD28 and CD45RA cell surface expression, and did not identify truly senescent and functionally exhausted cells. It is also difficult to rule out a delayed effect of exercise, or possible accumulation of very small effects with repeated exercise bouts. Thus, a more reliable way to address this question might be to use cell-labelling techniques to track the kinetics of individual T cells in vivo over the hours, days, weeks, months and years following exercise (Liu and Li 2014; Wagstaff et al. 1981; Westera et al. 2015). If different populations of late-stage differentiated T cells, some proven to be functional, and others proven to be exhausted, were labelled differently and infused into a volunteer, if both populations were present in blood samples pre- and post-exercise, then the selective “deletion” of cells would seem unlikely.

Assuming there is a post-exercise production of naïve cells, a key question is whether thymopoeisis, extrathymic T cell development, or a combination of the two is responsible. A way forward in discerning the possible contribution from each process might be to examine post-exercise changes in naïve T cell numbers in individuals who have been thymectomised during childhood compared to controls with an intact thymus (Sauce et al. 2009). If for example, a post-exercise surge in naïve T cell numbers was observed in controls, but not in individuals lacking a thymus, then it might be concluded that the process of extrathymic T cell development does not play an important role.

More broadly, in order to further examine possible anti-immunosenescence effects of exercise, studies should compare individuals across the full spectrum of exercise profiles (e.g., sedentary, inactive and active individuals, along with endurance athletes and extreme exercisers, see Fig. 1). These studies should objectively measure physical activity, directly measure cardiorespiratory fitness, and control for body composition assessed using precise methodology (e.g., dual-energy X-ray absorptiometry; DEXA). Studies should also examine a broader range of biomarkers associated with an ageing immune system (e.g., the numbers and proportions of B cells, their phenotype and function have been neglected so far) in addition to whole-body measures of immune competence, such as humoral and cell-mediated responses to novel antigens in vivo (Pascoe et al. 2014). Moreover, longitudinal exercise-training studies, undertaken in individuals exhibiting several hallmarks of an ageing immune system, perhaps with similar characteristics to the IRP, would reveal whether exercise can indeed rejuvenate aged immune profiles.

## A focus for future research: could oxidative stress induced by very high volume exercise result in pro-immunosenescence effects?

Further investigations are required to examine whether very high volumes of strenuous exercise undertaken over substantial periods of the lifespan exacerbate immunosenescence. One topic that might provide insight into mechanisms underlying anti- or pro-immunosenescence effects of exercise is redox balance. It is commonly cited that there is a linear relationship between reactive oxygen species production and the volume and intensity of exercise (Finaud et al. 2006). However, emerging evidence suggests that the relationship across the exercise continuum, as with infection risk, is likely to be J-shaped. Sedentary lifestyles, characterised by inactive skeletal muscle, sarcopenia and adipose tissue accumulation, are associated with moderate-to-high levels of reactive oxygen species, which can deplete antioxidant defences resulting in oxidation of proteins, lipids and DNA (Bjork et al. 2012; Derbre et al. 2014; Gratas-Delamarche et al. 2014; Le Lay and Simard 2014). Regular moderate-intensity exercise stimulates *optimal* reactive oxygen species production above resting values, but not to the levels associated with chronic sedentary behaviour (Bjork et al. 2012; Derbre et al. 2014; Gratas-Delamarche et al. 2014; Le Lay and Simard 2014). Optimal reactive oxygen species production is critical for cell function, especially immune cells, and the levels produced during moderate exercise have been proven to bring about beneficial adaptations in a number of cell types (Ji et al. 2006; Powers and Jackson 2008; Radak et al. 2008). At the extreme end of the exercise continuum, very prolonged and/or intensive bouts of exercise result in substantial oxidative stress (Knez et al. 2006). For example, the “oxidative footprint” of ultra-endurance exercise, is detectable in some cells of the immune system for weeks, if not months (Turner et al. 2011, 2013).

In addition to the clear relationships between redox balance and exercise, several lines of evidence suggest that oxidative stress is implicated in aspects of immunosenescence. For example, it has been shown that individuals over the age of 60 years, with a CD4:CD8 ratio <1.0, exhibit high levels of plasma advanced oxidation products, and high plasma antioxidant capacity—indicative of a compensatory “antioxidant response” to oxidative stress (Muller et al. 2015). Further, healthy individuals infected with CMV exhibit elevated biomarkers of oxidative stress (Lee et al. 2014), and CMV reactivation occurs frequently in autoimmune conditions characterised by chronic redox imbalance (Su et al. 2007). Mechanistic studies show that reactive oxygen species and one of the final products of lipid peroxidation, 4-hydroxy-2-nonenal (HNE), can directly reactivate CMV, but this process can be inhibited by antioxidants (Cinatl et al. 1999; Jaganjac et al. 2010; Speir 2000; Vossen et al. 1997). Finally, oxidative stress might promote CMV reactivation indirectly, considering that redox balance is intricately involved in cell-mediated immunity. For example, antigen processing and presentation is impaired by oxidative stress (Martner et al. 2011; Preynat-Seauve et al. 2003) and high levels of reactive oxygen species in T cells impair cytotoxicity, perhaps mediated by T cell lipid peroxidation, resulting in failure to control viruses (Henson et al. 2014; Matsushita et al. 2015). Finally, if antioxidant molecules are depleted in T cells, then cytokine production, can become uncontrolled (Michalek et al. 2012), potentially resulting in an inflammatory environment that is also known to reactivate CMV (Docke et al. 1994; Prosch et al. 1995; Stein et al. 1993). It is currently unknown whether very strenuous or prolonged exercise, which can result in oxidative stress, causes CMV reactivation—a process that might be a direct or indirect effect of exercise (Simpson et al. 2016; Turner et al. 2014a).

Although strong links have been shown between antioxidant supplementation and T cell function at rest (Hoffmann et al. 2010; Malmberg et al. 2002; Marthandan et al. 2013, 2014) the evidence for dietary antioxidants modulating exercise-induced changes in T cell function is less clear. For example, although self-reported illness symptoms following a period of demanding exercise training have been shown to be lower in athletes supplementing their diet with Quercetin, this antioxidant had no effect on assessments immune function before and after acute exercise (Nieman et al. 2007). Several other studies have reported that dietary antioxidants do not affect the typical changes in lymphocyte function induced by acute exercise, but many of these studies have examined non-specific aspects of cell-mediated immunity (McAnulty et al. 2011; Petersen et al. 2001). Encouragingly however, a murine study has shown that *N*-acetyl-cysteine prevents thymocyte apoptosis and cell loss caused by exercise-induced oxidative stress (Quadrilatero and Hoffman-Goetz 2005).

On the basis of evidence showing that exercise can positively and negatively influence immune function, evidence that exercise can disrupt redox homeostasis, and evidence implicating oxidative stress in immune deregulation, future studies investigating interaction between exercise, redox balance and immunosenescence seem warranted.

## Conclusion

Considering that ageing results in deleterious alterations to innate and adaptive immunity, and that exercise is a powerful stimulus of immune function, it is appealing that some forms of exercise might prevent and/or delay immunosenescence, perhaps even restoring immunosenescent profiles. A framework has been put forward providing a potential mechanism for how exercise might impact upon T cell immunosenescence. This hypothesis proposes that exercise mobilises late-stage differentiated T cells into the bloodstream, facilitating their extravasation to the tissues, resulting in a selective apoptosis of senescent/exhausted cells that occurs in parallel with increased thymic or extra-thymic output of naïve cells (Simpson 2011; Simpson and Guy 2009). While some ideas proposed in this “exercise makes immunological space” hypothesis are supported by robust evidence, other themes are contentious and have been hotly debated. However, a possible anti-immunosenescence effect of exercise could also be elicited indirectly (e.g., better anti-viral control, mediated by redox balance and less inflammatory activity, possibly linked to adipose tissue regulation). The limited evidence to date for exercise preventing, delaying or restoring T cell immunosenescence suggests there is a U-shaped relationship with categories of exercise behaviour (e.g., sedentary, inactive, active, very active, and extremely active; see Fig. 1). In addition, it is likely that in the context of ageing, it is the cumulative effect of behaviour, over a substantial period of the lifespan, which influences the rate or degree of immunosenescence.

## Footnotes

1 In this review, the term “exercise” refers to physical activities that are planned, structured, repetitive and undertaken for the purpose of improving or maintaining components of physical fitness and/or sporting performance. In places, individuals are referred to as being “active” or “inactive” due to convention in the literature. These descriptions infer that individuals undertake (or fail to undertake) a defined level of exercise (e.g., age-specific physical activity guidelines, such as those published by the World Health Organisation). The term “physical activity” includes leisure-time, occupational, home-based and transport-related activities. Although structured exercise is a component of physical activity (within the leisure-time domain), the other forms of physical activity are not considered in this article.
